# Supplementary material for: The relationship between home modifications and frailty among older adults: a scoping review protocol
Source: PLoS One. 2025 Nov 6;20(11):e0335822. doi: 10.1371/journal.pone.0335822 (PMC12591441; doi:10.1371/journal.pone.0335822)
Supplement: Attachment — Submitted filename: Response to reviewers.docx [file pone.0335822.s003.docx]

Response to Reviewers (point-by-point):

1. Please ensure that your manuscript meets PLOS ONE's style requirements, including those for file naming.

**Response: Checked**

2. Thank you for stating the following financial disclosure:

“The author MK serves as the project lead of the Housing and Digitalisation Competence Center Mecklenburg-Vorpommern (Landesfachstelle für Wohn- und Digitalisierungsberatung), a publicly funded initiative. The project is funded by the Ministry of Social Affairs, Health and Sport of Mecklenburg-Vorpommern. No additional financial support from private or commercial entities was received in relation to this work.”

**Response: We added: "The funders had no role in study design, data collection and analysis, decision to publish, or preparation of the manuscript."**

**We included this statement in our revised cover letter and in the manuscript.**

3. Thank you for stating the following in the Competing Interests section:

**Response: We added the statement: "This does not alter our adherence to PLOS ONE policies on sharing data and materials.” in our updated Competing Interests statement in our cover letter.**

4. When completing the data availability statement of the submission form, you indicated that you will make your data available on acceptance. We strongly recommend all authors decide on a data sharing plan before acceptance, as the process can be lengthy and hold up publication timelines. Please note that, though access restrictions are acceptable now, your entire data will need to be made freely accessible if your manuscript is accepted for publication. This policy applies to all data except where public deposition would breach compliance with the protocol approved by your research ethics board. If you are unable to adhere to our open data policy, please kindly revise your statement to explain your reasoning and we will seek the editor's input on an exemption. Please be assured that, once you have provided your new statement, the assessment of your exemption will not hold up the peer review process.

**Response: We are committed to adhering to the open data policy. As this is a protocol, no primary data have been collected yet, so there are no datasets to share at this stage.**

5. If the reviewer comments include a recommendation to cite specific previously published works, please review and evaluate these publications to determine whether they are relevant and should be cited. There is no requirement to cite these works unless the editor has indicated otherwise.

**Response: not applicable**

6. Please review your reference list to ensure that it is complete and correct. If you have cited papers that have been retracted, please include the rationale for doing so in the manuscript text, or remove these references and replace them with relevant current references. Any changes to the reference list should be mentioned in the rebuttal letter that accompanies your revised manuscript. If you need to cite a retracted article, indicate the article’s retracted status in the References list and also include a citation and full reference for the retraction notice.

**Response: checked**


Reviewers' comments:

Reviewer's Responses to Questions

**Comments to the Author**

Reviewer #1: The methodological proposal is solid, coherent, and aligned with the main conceptual frameworks and international guidelines for scoping reviews, such as the Arksey and O'Malley model, the Joanna Briggs Institute (JBI) guidelines, and the PRISMA-ScR checklist. The proposed methodology is adequate to meet the stated objectives and has high potential to generate relevant and useful evidence. The objectives are clearly formulated and focus on mapping and synthesizing the available evidence on the topic of interest. The application of the PCC (Population, Concept, Context) framework allows for an appropriate and relevant selection of studies. It is recommended that the protocol explicitly state the expected limitations, such as the possible heterogeneity among the included studies (in terms of design, intervention, and measurement of frailty). In addition to including the search time frame. It would be relevant to incorporate a section addressing the potential use of the findings for the design of home-based interventions and for the formulation of public policies.

**Response: We appreciate the reviewer’s thoughtful suggestions.**

**Ad Limitations: We have revised the protocol to explicitly outline the expected limitations of our review, particularly the potential heterogeneity among included studies. We have added a new “Limitations” section to the protocol.**

**Ad Search Time Frame: We have now clearly stated the time frame for the literature search in the “Search Strategy” section. The review will include studies published up to September 2025.**

**Ad Implications for Practice and Policy: we expanded the part on this in the “Background and Rationale” section.**

Reviewer #2: The Article was presented in a comprehensive, yet concise manner, the aims and objectives well put forward, Above all- a Great scoping review.

**Response: Thank you.**

Reviewer #3: Thank you for sending this article in its protocol version; here are some comments:

Methods:

Consider the protocol log.

**Response: We appreciate this recommendation**. **We have pre-registered this project on Open Science Framework (OSF) [****osf.io/6xajk]. Any amendments to the protocol will be documented there (= protocol log) and reported transparently in the final publication.**

It would be important to detail the search strategy and/or keywords to use.

**Response: We appreciate this recommendation. We have now included a more detailed (preliminary) search strategy in the Methods section of the protocol, including example keywords and Boolean operators.**

Additionally, it is important to define which type of test will be used to measure frailty, whether home modifications or accessibility will be evaluated in relation to frailty prevention and management outcomes, and how the frailty variable will be grouped or categorised for analysis.

**Response: Thank you for this feedback. As this is a scoping review, we do not aim to restrict the included studies to specific frailty assessment tools. However, we have clarified in the protocol that we will extract and report the** **type of frailty assessment used in each study.**

**We have also clarified that we will chart whether the study focuses on assessments of environmental accessibility home modifications, accessibility interventions, or both, and how these are linked to frailty prevention, mitigation, or management outcomes.**

It is important to include PRISMA-P as supplementary material because, although it was designed for systematic reviews, it is a protocol checklist that could be used for scoping reviews.

**Response:**
**Thank you for this suggestion. We have completed the PRISMA-ScR (Preferred Reporting Items for Systematic reviews and Meta-Analyses extension for Scoping Reviews) Checklist checklist and included it as a** **Supplementary File**.
